# Transparent, Photothermal, and Icephobic Surfaces via Layer‐by‐Layer Assembly

**DOI:** 10.1002/advs.202105986

**Published:** 2022-03-11

**Authors:** Shuwang Wu, Zhenyu Liang, Yupeng Li, Sarah Chay, Zhiyuan He, Sicong Tan, Jianjun Wang, Xinyuan Zhu, Ximin He

**Affiliations:** ^1^ Department of Materials Science and Engineering University of California Los Angeles CA 90095 USA; ^2^ School of Chemistry and Chemical Engineering State Key Laboratory of Metal Matrix Composites Shanghai Jiao Tong University 800 Dongchuan Road Shanghai 200240 China; ^3^ Key Laboratory of Green Printing Institute of Chemistry Chinese Academy of Sciences Beijing 100190 China; ^4^ School of Materials Science and Engineering Beijing Institute of Technology Beijing 100081 China; ^5^ Heat and Mass Transfer Center Institute of Engineering Thermophysics Chinese Academy of Sciences Beijing 100190 China

**Keywords:** icephobic, icing, layer‐by‐layer, photothermal, transparency

## Abstract

Icing and frosting on transparent surfaces compromise visibility on various optical equipment and transparent infrastructures. It remains challenging to fabricate energy‐saving coatings for harvesting solar energy while maintaining high transparency. Here, transparent, photothermic, and icephobic composite surfaces composed of photothermal nanomaterials and polyelectrolytes via layer‐by‐layer assembly are designed and constructed. The positively‐charged polypyrrole nanoparticles and negatively‐charged poly(acrylic acid) are assembled as exemplary materials via electrostatic attractions. The optically transparent photothermal coatings are successfully fabricated and exhibited photothermal capabilities and light‐transmittance performance. Among the various coatings applied, the seven‐bilayer coating can increase the temperature by 35 °C under 1.9‐sun illumination, maintaining high transmittance (>60%) of visible light. With sunlight illumination at subzero temperatures (> −35 °C), the coatings show pronounced capabilities to inhibit freezing, melt accumulated frost, and decrease ice adhesion. Precisely, the icephobic surfaces remain free of frost at −35 °C as long as sunlight illumination is present; the accumulated frost melts rapidly within 300 s. The ice adhesion strength decreases to ≈0 kPa as the melted water acts as a lubricant. Furthermore, the negatively‐charged graphene oxide and positively‐charged poly(diallyldimethylammonium chloride) show their material diversity applicable in the coating fabrication.

## Graphical abstract

The optically transparent photothermal coatings composed of photothermal nanomaterials are successfully fabricated via layer‐by‐layer assembly. With sunlight illumination at subzero temperatures (>−35 °C), the coatings show pronounced capabilities to inhibit freezing, melt accumulated frost, and decrease ice adhesion. It is a simple, low‐cost, and energy efficient strategy to make coatings for solving optical failure caused by icing/frosting.

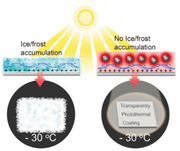

## 1 Introduction

Undesirable icing is a ubiquitous and unavoidable phenomenon that has been a long‐standing problem and disruption in human society.^[^ ^1^, ^2^ ^]^ Designing efficient anti‐icing/deicing materials has been in high demand in broad application on vehicles,^[^ ^3^ ^]^ aircraft,^[^ ^4^ ^]^ wind turbines,^[^ ^5^ ^]^ residential houses,^[^ ^6^ ^]^ power lines,^[^ ^7^ ^]^ and photovoltaics.^[^ ^8^, ^9^ ^]^ Till now, numerous strategies have been proposed to address the critical issues caused by frosting or icing. For example, superhydrophobic surfaces aim at removing the condensed water,^[^ ^10^ ^]^ while ion‐rich coatings target on depressing ice nucleation temperatures and delaying the freezing.^[^ ^11^, ^12^ ^]^ However, with these two kinds of strategies, unavoidably the surfaces would be eventually covered with frost/ice at extremely low temperatures. Therefore, tremendous efforts have been made to reduce the ice adhesion for removing accumulated frost/ice easily. Icephobic materials including hydrogel coatings,^[^ ^13^, ^14^, ^15^ ^]^ self‐repairing slippery surfaces,^[^ ^16^, ^17^ ^]^ and low‐interfacial toughness materials have been developed in this quest.^[^ ^6^, ^18^, ^19^ ^]^

Although significant efforts have been devoted to counteracting ice/frost growth and accumulation on all kinds of surfaces, some specific drawbacks in the strategies mentioned above remain, i.e., the fragileness of superhydrophobic surfaces,^[^ ^20^, ^21^ ^]^ the poor mechanical properties of ice nucleation depression, and low‐ice‐adhesion coatings, which limit their scalability and practical applications. Nowadays, anti‐icing strategies, most widely used in daily life, still rely on chemical, mechanical, and heating methods.^[^ ^22^, ^23^ ^]^ Unfortunately, these deicing methods suffer significantly from being low efficiency, environmentally unfriendly, or energy intensive. Deicing via electrical heating is still one of the most practical ways. However, the current heating methods require a specific design of the complex energy supply systems and are highly energy‐inefficient.^[^ ^22^ ^]^ Harvesting ubiquitous sunlight to heat the surfaces and removing frost/ice presents a promising way to address the existing heating problems posed by resistive heaters. Therefore, deice via utilizing sunlight is catching more and more attention. Recently, coatings composed of plasmonics,^[^ ^24^, ^25^, ^26^ ^]^ magnetic particles,^[^ ^27^, ^28^ ^]^ and carbon nanomaterials^[^ ^28^, ^29^, ^30^ ^]^ have shown the abilities to generate heat via the photothermal effect to remove accumulated frost/ice. However, there is a contradiction between utilizing sunlight for anti‐icing and maintaining transmittance in some vital fields, wherein optical malfunctions frequently result from the icing/frosting happening on telescope lens, windows, windshields, electronic displays, and solar cells.^[^ ^31^ ^]^ Especially, more and more buildings nowadays install giant glasses for better vision and beauty.^[^ ^32^ ^]^ Even though some inspiring works have been done,^[^ ^25^, ^33^, ^34^ ^]^ achieving outstanding anti‐icing ability through photothermal effect while maintaining high transmittance of visible light benefiting energy saving, raises a challenging need to be addressed urgently.

Herein, we propose to make optically transparent, photothermally icephobic surfaces via layer‐by‐layer (LBL) assembly. LBL has been a classic method commonly used to create functional coatings for various applications by assembling two oppositely charged materials through electrostatic attractions.^[^ ^35^, ^36^ ^]^ The structures and their thickness can be easily regulated, providing a facile way to balance the transmittance and photothermal effect. In this work, we chose positively charged polypyrrole (PPy) nanoparticles and negatively charge poly(acrylic acid) (PAA) as prototypical systems to make the transparent photothermal anti‐icing coatings. By tuning the layers of deposited PPy nanoparticles and PAA, the coatings exhibit the ability to convert solar energy into heat while maintaining high transmittance. Under sunlight illumination, the coating of seven LBL bilayers can increase the temperature by 35 °C while maintaining >60% transmittance of visible light. With sunlight illumination at subzero temperatures, the coatings show multiple pronounced abilities to inhibit freezing, melt accumulated frost, and decrease ice adhesion. Additionally, the coatings successfully made of other LBL building blocks, such as the negatively charged graphene oxide and positively charged poly(diallyldimethylammonium chloride) (PDDA), demonstrated the broad choices and diversity of materials for the modular‐design coating fabrication. The simplicity, low‐cost, material diversity, and energy saving of the coatings via LBL assembly render this process a great promise and benefits for practical anti‐icing applications.

## 2 Results and Discussion

### 2.1 Design of Transparent Icephobic Coatings

**Figure 1:**
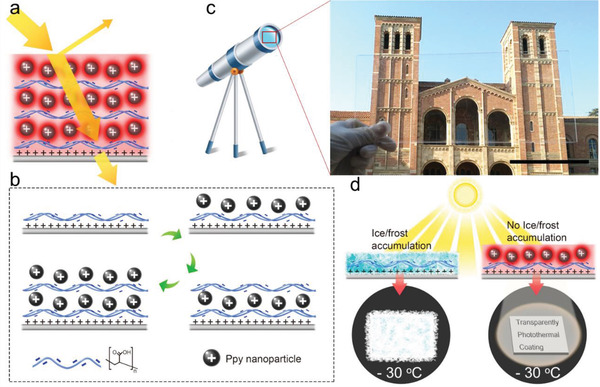
Schematic diagrams of the transparent icephobic coatings. a) Schematic diagrams of sunlight absorption, transmission, and reflection. b) The assembly processes of layer‐by‐layer assembly. c) The application scenario and a transparent large‐sized sample. d) Schematic illustration of the anti‐icing ability of the coating under sunlight illumination. Scale bar in (c) = 10 cm.

The coatings via LBL assembly were proposed to solve the contradictions between transmittance and photothermal effect. As shown in **Figure** **1**a, the coating is composed of PPy nanoparticles and polymers. Most of the incident sunlight could pass through the coating, guaranteeing the transmittance, while a part of the sunlight was absorbed by the PPy nanoparticles for generating heat. The remaining small fraction of sunlight was reflected. The transparent substrates were initially modified with positively charged chemicals (Figure S1, Supporting Information). Subsequently, the coatings were fabricated with negatively charged PAA and positively charged PPy nanoparticles via LBL assembly. The number of layers and the thickness of the coating could be simply tuned by the cycles of processing. The coating fabrication is highly scalable due to the nature of solution‐based processing. As demonstrated in Figure 1c, a large glass slide coated with nanoparticles had high transmittance, and through which the building can be seen very clearly. With the transparent photothermal coating, the surface would stay frost/ice‐free under sunlight and maintain transparency at subzero temperatures (Figure 1d).

### 2.2 Assembly of Coatings

**Figure 2:**
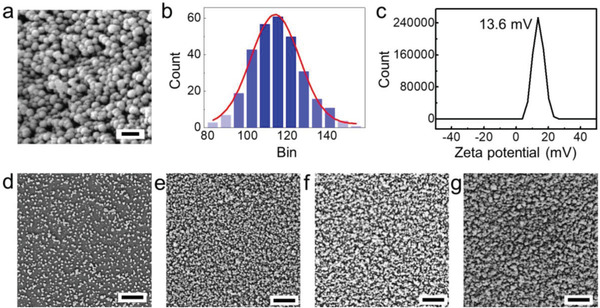
Characterization of the as‐synthesized PPy nanoparticles and the coatings. a) SEM image of the PPy nanoparticles. b) The size distribution of the nanoparticles. c) The zeta potential of the PPy nanoparticles. d–g) The SEM images of the coatings of 1, 3, 5, and 7 bilayers. Scale bar in (a) = 200 nm, and in (d–g) = 1 µm.

The PPy nanoparticles were synthesized in a 1 wt% PVA solution, in which the PVA acted as a surfactant helping with the stable dispersion of PPy nanoparticles (Figure S2, Supporting Information). As shown in **Figure** **2**a,b, the as‐synthesized PPy nanoparticles display a uniform size range from 80 to 150 nm with an average size of 125 nm. The backbone of PPy is positively charged (Figure S2, Supporting Information)^[^ ^37^ ^]^ making the PPy nanoparticles positively charged which was demonstrated with the zeta potential (Figure 2c), while PAA was negatively charged (Figure S3, Supporting Information). After the LBL assembly process, the nanoparticles were successfully coated onto the glass substrates. As shown in Figure 2d–g, the PPy nanoparticles were distributed uniformly on the surface, and the densities of nanoparticles increased as the number of bilayers increased from 1 to 7. The average coating thickness of a seven‐layer surface was 1 µm .

### 2.3 Characterizations of Transmittances and Temperatures

**Figure 3:**
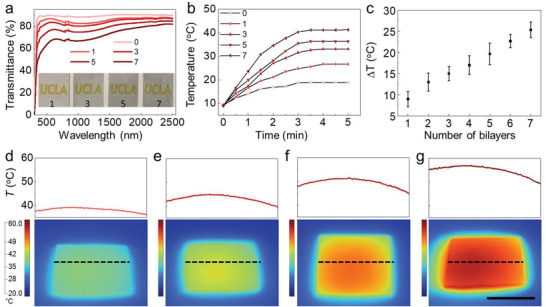
The transmittances and temperatures of the transparent icephobic coatings. a) The spectrum of coatings with 1, 3, 5, and 7 bilayers. b) The temperature versus illumination time under 1.9 sun illumination (including the internal reflection of 90% in the insulating chamber under the 1 sun illumination of a solar simulator). c) Temperature increase (*∆T*) for coatings with different numbers of bilayers. d–g) Infrared camera images and the temperature profiles of coatings with 1 (d), 3 (e), 5 (f), and 7 (g) bilayer(s) after 5 min illumination under 1.9 sun illumination. The temperature profiles represent the temperatures across the dashed lines. Scale bar = 1 cm.

As the numbers of bilayers increased, a decrease was observed in the corresponding transmittance values. As shown in **Figure** **3**a, across the full spectrum ranging from 300 to 2500 nm, all the transmittances remained at a high level for the visible light. However, the average transmittance decreased from 89% to 63% as the numbers of bilayers increased from 1 to 7 (Figure 3a; Figure S4, Supporting Information). The transmittances remained even higher in the infrared section as the numbers of bilayers increased (Figure 3a; Figure S4, Supporting Information). Meanwhile, the reflectance of the coating with various bilayers demonstrated a small fraction of light was reflected making all the reflectance lower than 10% (Figure S5, Supporting Information). The temperatures of coatings with various bilayers numbers were systematically measured after different illuminations times under 1.9 sun sunlight (1.9 kW m^−2^, including the internal reflection of 90% in the insulating chamber under the 0.26 sun to 1 sun illumination of a solar simulator) (Figure 3b; Figure S6, Supporting Information). The temperatures increased as the illumination times were prolonged and reached plateaus after the illumination time crossed 4 min (Figure 3b). The temperature also increased rapidly at low‐temperature (−20 °C) environments (Figure S7 and Movie S1, Supporting Information). The temperature increases of the coatings (∆*T*, excluding a base temperature increase ∆*T*_base_ = 9.5 °C as measured, which the contribution from the heat generated by the light in air) with different bilayers measured under 1.9 sun illumination were plotted in Figure 3c. It showed that the ∆*T* increased from 9.4 to 25.3 °C, as the number of bilayers increased from 1 to 7. Furthermore, the Δ*T* of the seven‐bilayer coating has been measured after exposure to different light intensities. It increased from 5.6 to 25.3 °C, as the overall solar intensity increased from 0.5 to 1.9 sun (including the internal reflection of 90%, under the 0.26 sun to 1 sun illumination of a solar simulator) (Figure S8, Supporting Information). The infrared images presented the temperature distributions on the coatings and the surrounding uncoated surfaces under 1.9 sun illumination (Figure 3d–g). The temperatures profiles show that the temperatures at the edges of coated region (marked with dashed lines) were higher than that in the center of the coated region, which was attributed to the higher heat‐transfer rate of the edges. The simulation results captured higher temperature increases under 1.9 sun illumination, which might be due to the heat leakage during experiments (Figure S9, Supporting Information).

### 2.4 Anti‐Icing Performances

**Figure 4:**
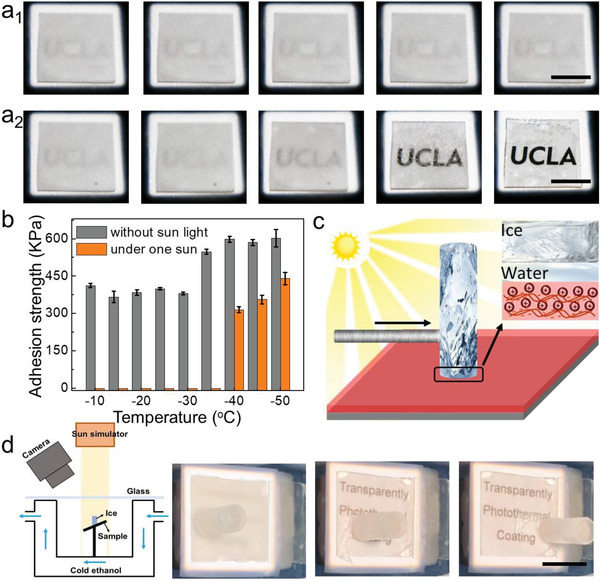
Anti‐icing abilities of the surfaces. a) Frost on the bare glass (a_1_) and the glass coated with seven bilayers (a_2_) under 1.9 sun illumination. b) The ice adhesion strengths on a glass slide with a seven‐bilayer coating with and without sunlight at different temperatures. c) The schematic mechanism of the low ice adhesion with sunlight illumination. d) The schematic of the experimental setup and the optical images of an ice pillar on a 30°‐tilted glass surface coated with seven bilayers at −20 °C. (a,d) Scale bars = 1 cm.

Ultimately, the anti‐icing properties of the coatings were studied and demonstrated. At −30 °C, the glass slides with a seven‐bilayer coating remained unfrozen because of the photothermal effect. In stark contrast, the bare glass slide was covered with frost and the words under the glass slide could not be observed clearly due to the frost blockage (Figure S10, Supporting Information). To observe the melting process under sunlight illumination, we monitored the bare glass slide and the glass slide coated with seven bilayers, after keeping them covered with frost at −20 °C for 1 h. As shown in Figure 4a1, the frost on the bare glass slide remained frozen, and the word “UCLA” underneath the glass cannot be viewed clearly. On the contrary, the frost melted rapidly within 300 s on the seven‐bilayer coated glass slide (**Figure** **4**a2; Movie S2, Supporting Information). To further investigate the deicing ability, the adhesion strengths of ice on the glass slides with a seven‐bilayer coating were tested at different subzero temperatures, with and without sunlight illumination (Figure S11, Supporting Information). When the temperature was equal to or higher than −35 °C, the ice adhesion strengths were much lower under 1.9 sun illumination than that without sunlight. For example, at −35 °C, the ice adhesion was 580 kPa, and once it was exposed to sunlight for 5 min, the ice adhesion significantly decreased to ≈0 kPa (Figure 4b).^[^ ^5^, ^16^, ^19^ ^]^ Such an ultralow adhesion resulted from the fact that under sunlight, the ice in contact with the substrates melted, upon the effective local heating via the photothermal effect, and consequently the thin layer of water at the interface of the substrate and bulk ice acted as a lubricant, reducing the ice adhesion to ≈0 kPa (Figure 4c).^[^ ^15^, ^38^ ^]^ As the temperature was lower than −35 °C, the heat generated through the photothermal effect became less sufficient to melt the ice. Therefore, the ice adhesion increased apparently. However, the ice adhesion strengths were still substantially lower than those without sunlight at the same temperature.

To further demonstrate the remarkable ability to decrease ice adhesion strength with sunlight illumination at subzero temperatures. An ice pillar was frozen on the glass slide with a seven‐bilayer coating at −20 °C for 30 min; subsequently, the glass slides were tilted to 30° and illuminated with sunlight (Figure 4d). The temperatures of air, the ice pillar and the sample were measured with three thermal probes. After shinning sunlight for 10 min, the surface temperature increased to 3.7 °C due to the photothermal effect, and the ice pillar readily slid off the surface while remaining frozen (Figure 4d; Movie S3, Supporting Information). However, the temperatures of the air and ice pillar were −15.3 and −14.1 °C, respectively. The LBL assembly is known as a classic universal method for fabricating composite coatings of large varieties of compositions and properties. Therefore, many photothermal nanomaterials such as carbon nanotubes, carbon nanodots, gold nanoparticles, and polymers can be made as photothermal icephobic coatings. Among broad choices of building blocks for this generalizable coating design, here we did further demonstration with the negatively charged graphene oxide and positively charged PDDA. They were assembled onto the surface of the glass slides (Figure S12, Supporting Information) and tested. After the deposited graphene oxide was reduced to graphene, the coatings had a pronounced photothermal effect while preserving relatively high transmittances (Figure S13, Supporting Information).

## 3 Conclusion

In summary, transparent, photothermal, and icephobic surfaces have been successfully constructed via the LBL assembly technique. The positively charged PPy nanoparticles, which can transfer sunlight into heat via photothermal effect and negatively charge PAA, were used as prototypical materials to make transparent photothermal coatings. By tuning the layers of deposited PPy nanoparticles and PAA, the coatings were made to exhibit the ability to transfer solar energy into heat while maintaining high transmittance. Under sunlight illumination, the coating of seven bilayers can increase the temperature by 35 °C and maintain >60% of the transmittance. The coatings show pronounced inhibition of freezing, melting accumulated frost, and decreasing ice adhesion with sunlight illumination at subzero temperatures. The use of negatively charged graphene oxide and positively charged PDDA demonstrate the diversity of the materials. The simplicity, low‐cost, material diversity, and energy efficiency of the coatings fabricated via LBL assembly make it a promising and beneficial strategy for solving optical failure caused by icing/frosting.

## 4 Experimental Section

### Materials

Poly(vinyl alcohol) (molecular weight = 89 000–98 000, hydrolysis degree = 99%, Sigma), pyrrole (reagent grade, 98%, Sigma), iron(III) chloride hexahydrate (Sigma), poly(acrylic acid), poly(diallyldimethylammonium chloride) (PDDA) (Mw = 200–350 kDa, from Aldrich), (3‐aminopropyl)tris(trimethylsiloxy)silane (Sigma, ≥95%) milli‐Q water. All the materials were used without further purification.

### Preparation of Graphene Oxide

The graphene oxide was synthesized following the report work.^[^ ^39^ ^]^ Briefly, 1 g graphite powder was added slowly and stirred in 25 mL 98% H_2_SO_4_ for 3 h. Afterward, 2 g KMnO_4_ was gradually added and the temperature was kept below 20 °C with an ice bath. The mixture was further stirred at 20 °C (kept in an ice bath) for 3 h and diluted with 100 mL water under vigorous stirring (the water was slowly added). 5 mL 30% H_2_O_2_ solution and 75 mL pure water were mixed and added slowly into the mixture. The suspension was washed with 5% HCl solution and then plenty of water until the pH was ≈7. Finally, the GO solution was dialyzed and freeze‐dried.

### Preparation of PPy Nanoparticles

The PVA precursor solution was first prepared. 1 g PVA was dissolved in 100 mL water and heated to 90 °C with magnetic stirring to form a transparent solution. After the PVA solution was cooled down to room temperature, 3.5 g iron(III) chloride hexahydrate was added into the solution. Afterward, 1 mL pyrrole was injected into the solution slowly with magnetic stirring (600 rpm). The solution turned black immediately and was kept overnight. Finally, the solution was dialyzed for the further usage.

### Fabrication of Icephobic Surfaces

The glass slides were first washed by acetone, IPA, and deionized water in this order. After drying, the substrates were treated with oxygen plasma and then immersed in the silane solution (100 mL deionized water, 10 µL of acetic acid with pH 3.5 and 2 wt% of TMSPMA) for 2 h. After the incubation, the substrates were washed with ethanol and completely dried. After that, the as‐prepared substrates were dipped in polyanion solutions (PAA, 1 wt%) and PPy dispersion alternatively (for 15 min) and rinsed by plenty of pure water. Dipping and rinsing steps were repeated until the desired layers were obtained. The GO/PDDA coatings were fabricated with similar processes and the samples were immersed HI solution (0.5%) for 2 h to reduce the GO. After reducing the GO, the samples were dried and stored for further characterizations.

### Characterizations

The morphology was observed with a Supra 40VP scanning electron microscope. Zeta potential was measured with Zetasizer Nano ZS ZEN3600. Sun simulator was used to provide light source and the temperature was monitored and recorded with a thermocouple. An infrared camera (Fluke XV) was used to map the temperature of the samples.

### Anti‐Icing Experiments

These experiments were proceeded in a homemade system (Appendix and Figure S12, Supporting Information). The IR images and videos were taken with an infrared camera (Fluke XV). The freezing and melting processes were recorded with a digital camera.

### Ice Adhesion Measurement

The ice adhesion was measured with the method in the previous report.^[^ ^40^ ^]^ The setup is customized consisting of a cooling stage, an *XY* motion stage, a force transducer, and a sample chamber (Figure S10, Supporting Information). Quartz was used to cover the chamber because of the maximum sunlight transmission. Nine samples were fixed on the cooling stage with the insulation foam (thickness: 3 mm) between the samples and the cooling stage to avoid rapid heat exchange. Ice columns were formed by injecting 1 mL water into the container. It was kept at the testing temperatures for 5 h to ensure the complete freezing of the ice columns. The nitrogen gas was purged into the chamber to minimize the frost formation. During the test, the force transducer approached toward the ice column at a speed of 0.5 mm s^−1^ and the force probe was kept as close to the sample surface as possible, ≈1 mm. The resolution of the force transducers was 0.01 N. The peak force for detaching the ice column was recorded. Each ice adhesion strength at different temperatures was averaged by 9 individual measurements. Ice adhesion measurements under one sun were performed with a solar illumination intensity of 100 mW cm^2^. The experiments of ice sliding with a setup were performed (Figure 4d). The cold ethanol was circulated around a chamber to cool the air in the chamber to mimic outdoor subzero environment. An ice pillar was frozen on the glass slide with a seven‐bilayer coating at −20 °C for 30 min; subsequently, the glass slides were tilted to 30°. The whole setup was kept for another 30 min for get a stable temperature (−20 °C) and illuminated with sunlight afterward. The temperatures of the air, ice pillar, and the sample were measured with three thermal probes.

### Modeling and Simulation

The heat transfer simulations were carried out with ANSYS FLUENT 6.3 software. In the modeling, the LBL coatings on glass with different numbers of bilayers were selected as objective systems to study the temperature distribution under 1 sun illumination. The samples were placed on a thermal insulation plank (5 mm thick) which was in a closed quartz chamber which temperature kept 243 K on upper and side wall. The light source was placed above the chamber to illuminate the samples. The incident angle is 0^o^ (*q*_i_ = 1.0 kW m^−2^). The length, width, and thickness of the samples are 25, 25, and 1 mm. The initial temperature of the model is set as 243 K.

The transmittance was speculated based on the experimental results. The insulation foam at the bottom of the samples absorbed 10% of the light after it went through the samples. Afterward, the remaining light was reflected backward and absorbed by the photothermal coatings again. Based on the aforementioned processes, the overall heat absorbed was calculated and the temperature distribution was obtained by applying the heat on the bottom of the glass substrates.

The whole simulation chamber is closed. The temperature of the upper and surrounding boundary was set as 243 K. The heat absorbed by the substrates (direct irradiated and reflected on photothermal coatings, insulation foam) was obtained based on the above‐mentioned calculations. The other parts of the system were set as thermally insulating.

The governing equations of airflow and temperature fields were shown in the following equations

(1) $$\rho_{0}\frac{dV}{dt}=\rho g-\nabla p+\mu \nabla^{2}V$$

(2) $$\rho_{0}C_{p}\frac{dT}{dt}=\lambda \nabla^{2}T+S$$

where *μ* is the air dynamic viscosity,*λ* is thermal conductivity, and *S* is the source item.

Here Boussinesq approximation was applied to calculate the natural convection of air, which the density of gravity term is

(3) $$\rho =\rho_{0}\left[1-\alpha \left(T-T_{0}\right)\right]$$

where *α* is expansion coefficient, set as 0.0027 1/k, *ρ*_0_ and *T*_0_ are reference density and temperature (−30 °C) respectively.

The solid film is thermal conductive inside and the substrate is heat‐insulating, which heat transfer can be expressed in the following equation

(4) $$\rho_{0}C_{p}\frac{\partial V}{\partial t}=\lambda \nabla^{2}T+q$$

The solar thermal effect, heat transfer, and temperature distribution on various substrates were studied. During the simulation, various parameters were included together to simulate the temperature distribution such as the transmittance of atomized droplets, the light absorption of the photothermal coating, the absorption and the reflection of the thermal insulating foam below the samples, and the air convection. The temperature distribution on glass obtained with experiments is consistent with the simulation result. For the samples with the same transmittance, temperature increases measured experimentally are slightly higher than the corresponding simulation results, which is possibly because of the heat leakage of the experimental system.

## Conflict of Interest

The authors declare no conflict of interest.

## Supporting information

Supporting Information

Supplemental Movie 1

Supplemental Movie 2

Supplemental Movie 3

## Data Availability

The data that support the findings of this study are available from the corresponding author upon reasonable request.
