# Supplementary material for: Transparent, Photothermal, and Icephobic Surfaces via Layer‐by‐Layer Assembly
Source: Adv Sci (Weinh). 2022 Mar 11;9(14):2105986. doi: 10.1002/advs.202105986 (PMC9108600; doi:10.1002/advs.202105986)
Supplement: Supplementary file 1 — Supporting Information [file ADVS-9-2105986-s004.pdf]

Supporting Information

Transparent, photothermal, and icephobic surfaces via layer-by-layer assembly

Shuwang Wu, Zhenyu Liang, Yupeng Li, Sarah Chay, Zhiyuan He, Sicong Tan, Jianjun Wang, Xinyuan Zhu*, Ximin He**

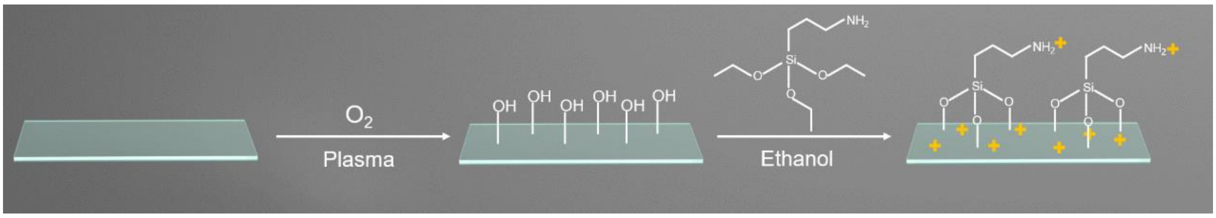

Figure S1. Modifying the substrates with siloxane to make it positively charged.

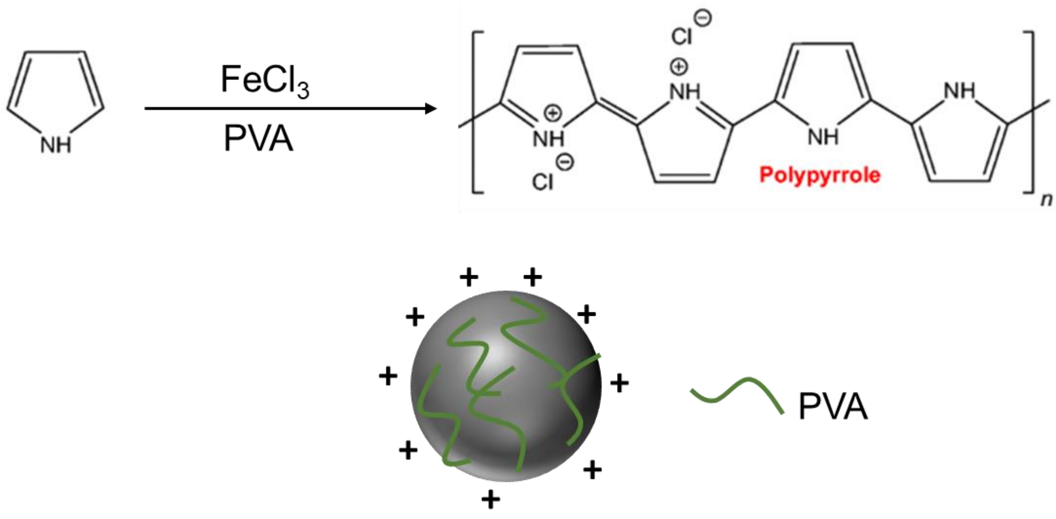

Figure S2. Synthesis of PPy nanoparticles. The as-synthesized nanoparticles were coated by PVA which helped with the stable dispersion.

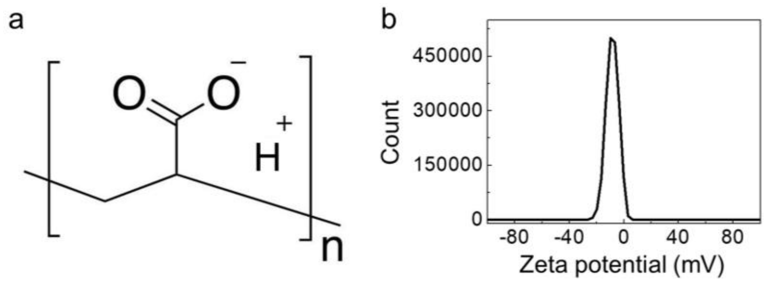

Figure S3. Zeta potential of PAA. The PAA concentration was 1 wt%.

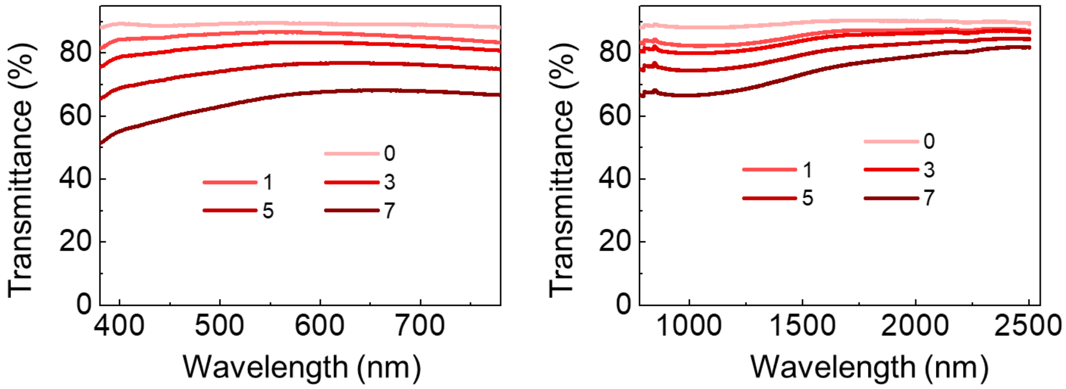

Figure S4. The transmittance of the PAA/PPy coatings of different numbers of layers at visible and IR light, respectively.

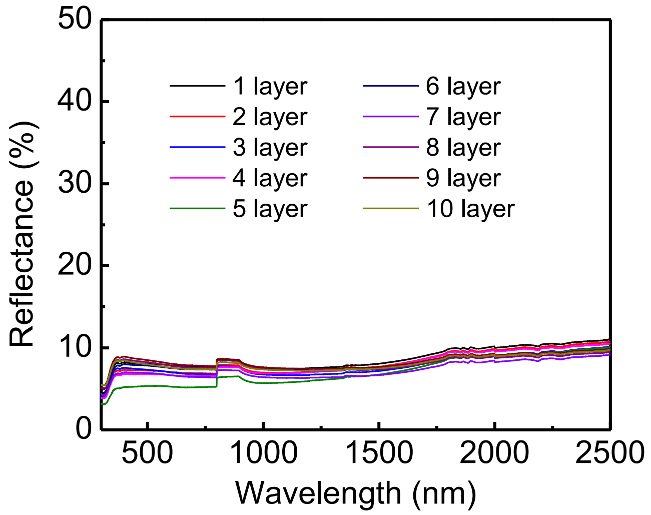

Figure S5. The reflectance of PAA/PPy coatings with various bilayers.

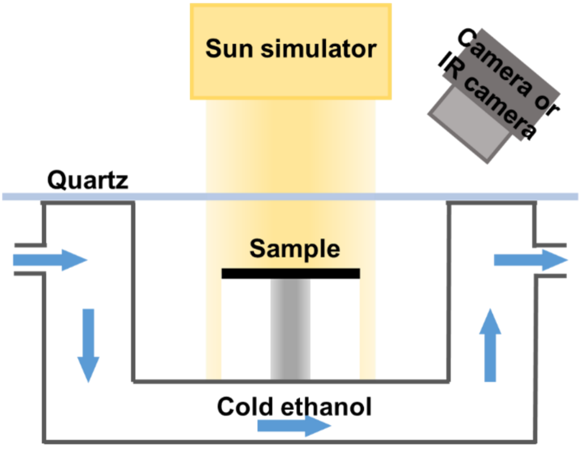

Figure S6. The experimental setup for the measurements of temperature of the samples.

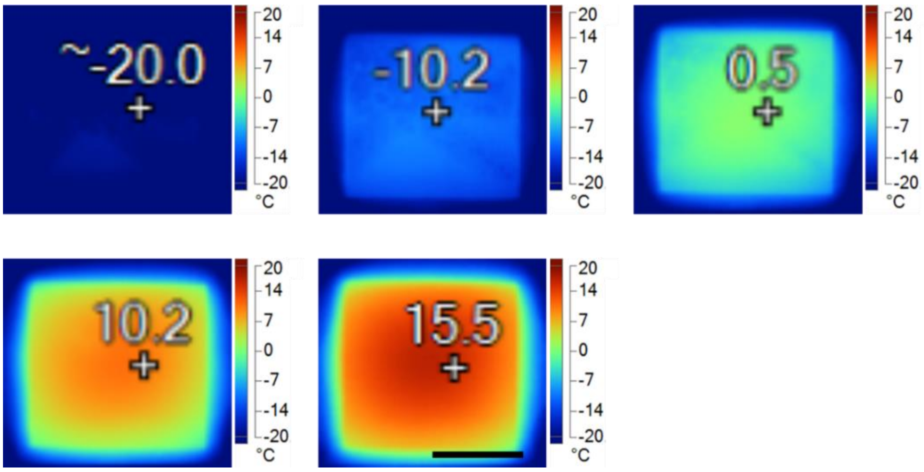

Figure S7. The IR images of sample with 7 bilayers under one sun. Scale bar = 1 cm.

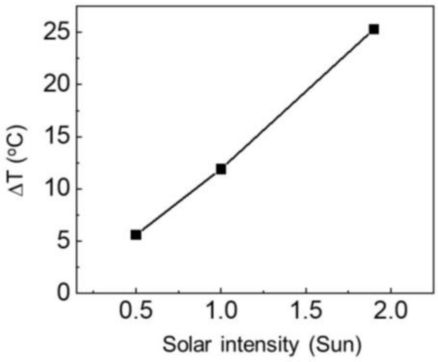

Figure S8. Temperature increases (ΔT) of coatings with 7 bilayers under different solar intensities.

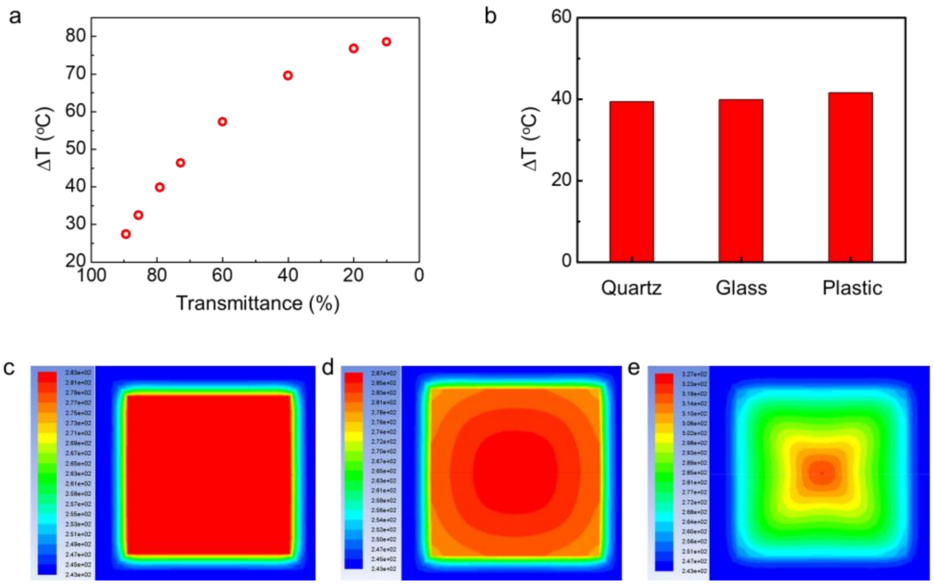

Figure S9. (a) Simulated temperature increases of samples with different transmittance (glass as the substrate). (b) Simulated temperature increases on different substrates when the transmittance is 79%. (c-d) The simulated temperature distribution on quartz, glass and plastic respectively.

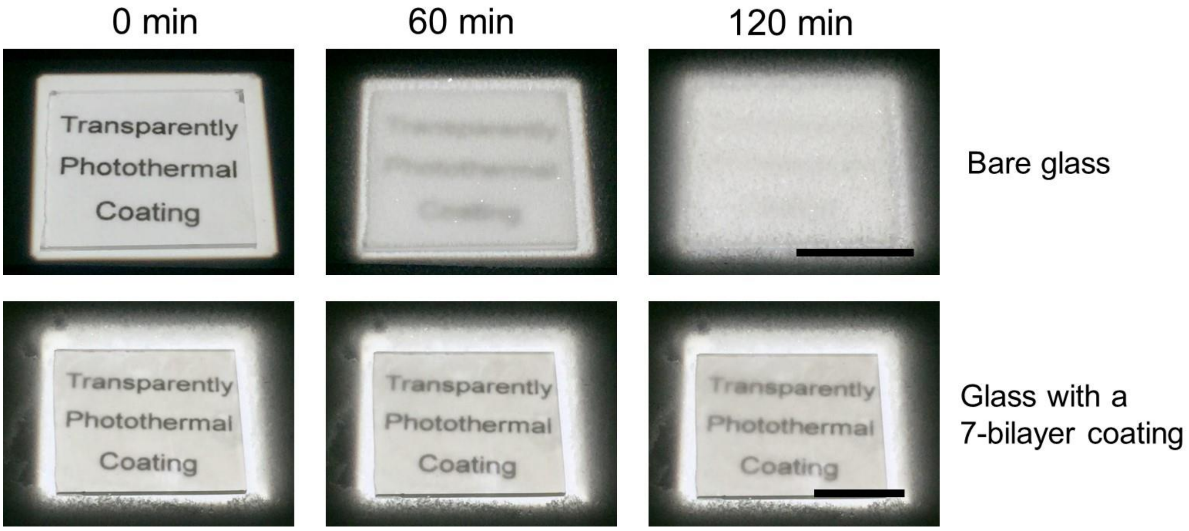

Figure S10. The bare glass and glass coated with 7 bilayers under 1.9 sun at -30 °C. Scale bars = 1 cm.

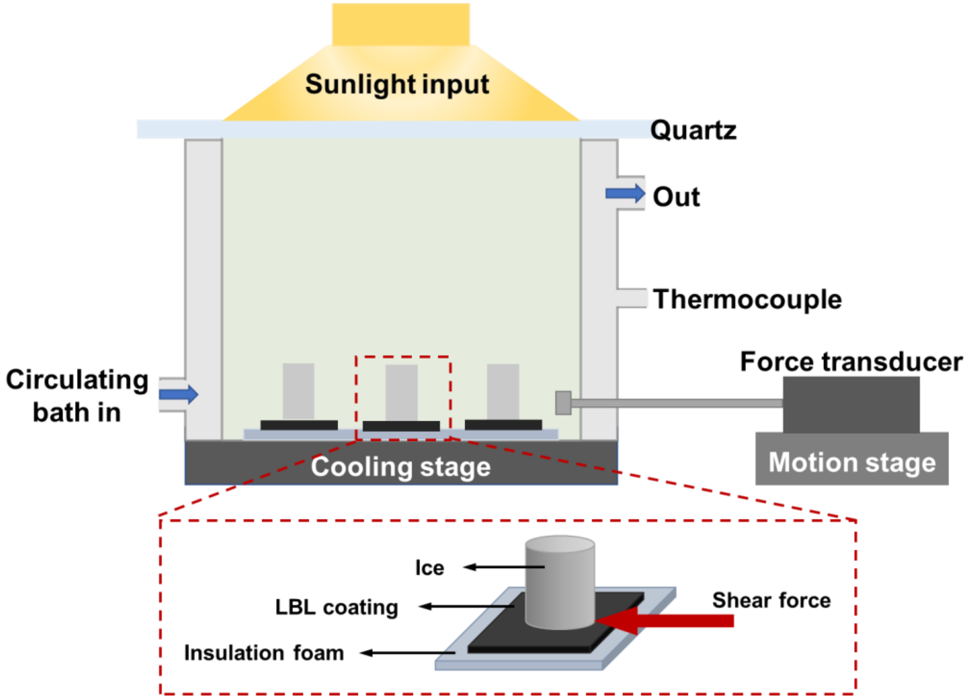

Figure S11. Schematic of ice adhesion measurement under sunlight illumination.

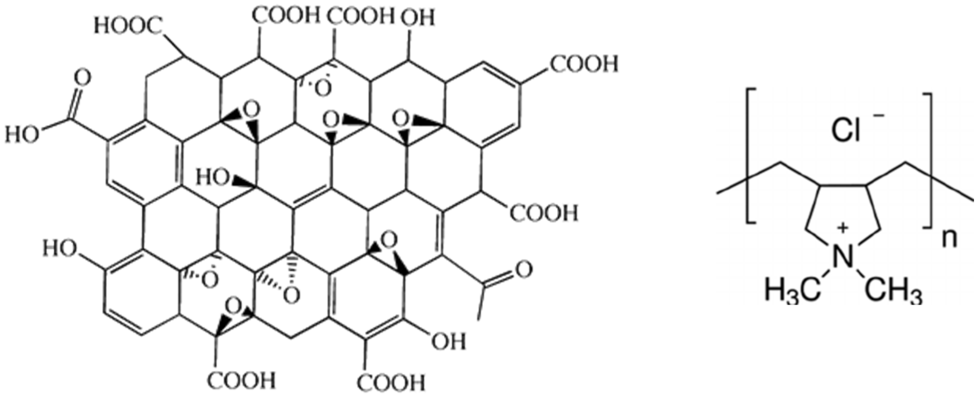

Figure S12. The chemical structures of graphene oxide and PDDA.

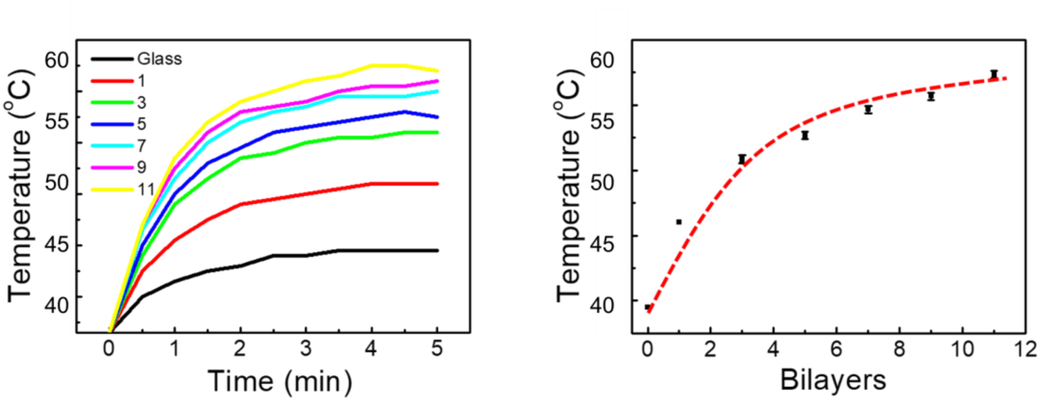

Figure S13. The temperatures of glass slides coated with different numbers of bilayers of graphene/PDDA under one sun.
